# 2-[(1*RS*,3*RS*,3a*RS*,6a*SR*)-5-Benzyl-4,6-dioxo-3-phenyl­octa­hydro­pyrrolo­[3,4-*c*]pyrrol-1-yl]acetamide

**DOI:** 10.1107/S1600536811045181

**Published:** 2011-11-05

**Authors:** Konstantin V. Kudryavtsev, Andrei V. Churakov, Ozdemir Dogan

**Affiliations:** a Department of Chemistry, M.V. Lomonosov Moscow State University, Leninskie Gory 1/3, Moscow 119991, Russian Federation; b Institute of General and Inorganic Chemistry, Russian Academy of Sciences, Leninskii prosp. 31, Moscow 119991, Russian Federation; c Department of Chemistry, Middle East Technical University, Ankara 06531, Turkey

## Abstract

In the title compound, C_21_H_21_N_3_O_3_, the relative stereochemistry of the four stereogenic C atoms has been determined. The dihedral angle between the phenyl rings is 77.63 (7)°. In the crystal, ribbons spread along the *a* axis are formed by N—H⋯O hydrogen bonds. C—H⋯π inter­actions also occur.

## Related literature

For general background to chemistry affording polycyclic pyrrolidine-based scaffolds, see: Kudryavtsev & Irkha (2005 ▶); Kudryavtsev (2008 ▶, 2011 ▶).
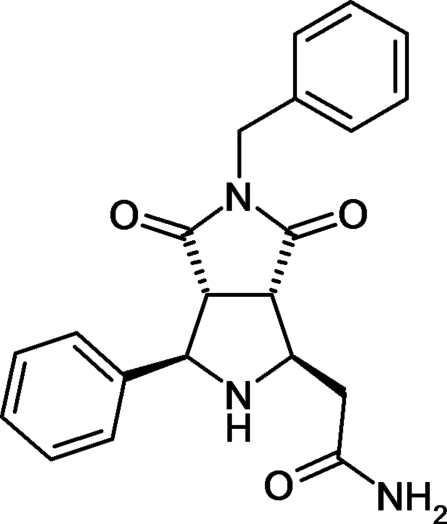


## Experimental

### 

#### Crystal data

C_21_H_21_N_3_O_3_
*M* *_r_* = 363.41
Triclinic, 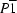

*a* = 9.101 (5) Å
*b* = 9.270 (5) Å
*c* = 12.945 (5) Å
α = 103.73 (4)°
β = 92.30 (4)°
γ = 113.94 (4)°
*V* = 958.1 (8) Å^3^
*Z* = 2
Mo *K*α radiation
μ = 0.09 mm^−1^
*T* = 293 K
0.50 × 0.40 × 0.30 mm

#### Data collection

Enraf–Nonius CAD-4 diffractometer
4399 measured reflections
3561 independent reflections
2309 reflections with *I* > 2σ(*I*)
*R*_int_ = 0.011
2 standard reflections every 120 min  intensity decay: none

#### Refinement

*R*[*F* ^2^ > 2σ(*F* ^2^)] = 0.035
*wR*(*F* ^2^) = 0.108
*S* = 1.02
3561 reflections
329 parameters
All H-atom parameters refined
Δρ_max_ = 0.23 e Å^−3^
Δρ_min_ = −0.16 e Å^−3^

### 

Data collection: *CAD4* (Schagen *et al.*, 1988 ▶); cell refinement: *CAD4*; data reduction: *XCAD4* (Harms, 1997 ▶); program(s) used to solve structure: *SHELXTL* (Sheldrick, 2008 ▶); program(s) used to refine structure: *SHELXTL*; molecular graphics: *SHELXTL*; software used to prepare material for publication: *SHELXTL*.

## Supplementary Material

Crystal structure: contains datablock(s) I, global. DOI: 10.1107/S1600536811045181/ff2039sup1.cif

Structure factors: contains datablock(s) I. DOI: 10.1107/S1600536811045181/ff2039Isup2.hkl

Supplementary material file. DOI: 10.1107/S1600536811045181/ff2039Isup3.cml

Additional supplementary materials:  crystallographic information; 3D view; checkCIF report

## Figures and Tables

**Table 1:** Hydrogen-bond geometry (Å, °) *Cg*1 is the centroid of the C10–C15 phenyl ring.

*D*—H⋯*A*	*D*—H	H⋯*A*	*D*⋯*A*	*D*—H⋯*A*
N3—H32⋯O1^i^	0.91 (2)	2.194 (19)	3.003 (2)	147.3 (16)
N3—H31⋯O3^ii^	0.90 (2)	2.00 (2)	2.903 (2)	175.7 (18)
C18—H18⋯*Cg*1^iii^	0.93 (2)	2.71	3.627	166.5

Symmetry codes: (i) 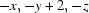; (ii) 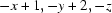; (iii) 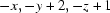.

**Fig. 1.**
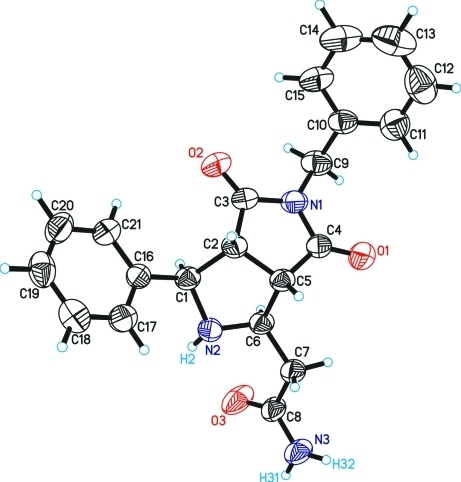
The molecular structure of the title compound, showing the numbering scheme adopted. Displacement ellipsoids are shown at the 50% probability level.

**Fig. 2.**
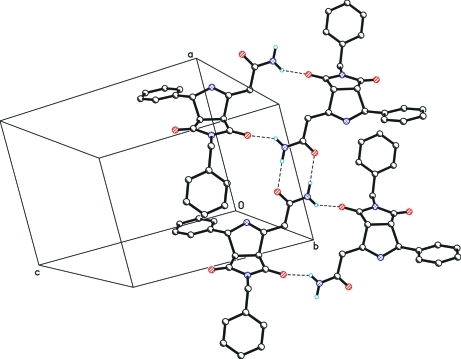
Hydrogen-bonded ribbons along a-axis in the structure of the title compound.

## supplementary crystallographic information

### Comment

The adjacent molecules are combined into ribbons along *a*-axis by hydrogen bonds between neighbouring amide groups (Fig. 2). These ribbons are linked by T-shaped C—H···π interactions between phenyl substituents. Of interest, secondary amine hydrogen atom N*H* does not participate in hydrogen bonding.

### Experimental

1.134 g (10.69 mmol) of benzaldehyde, 1.410 g (10.68 mmol) of *L*-asparagine and 2.000 g (10.68 mmol) of *N*-benzylmaleimide were stirred at 140°C in 30 ml of DMF under an inert atmosphere during 4 h. After cooling to room temperature the reaction mixture was concentrated on rotary evaporator. The residue was chromatographed on silica gel 60 (particle size 0.040–0.063 mm) using CHCl_3_—MeOH (40: 1) as eluent. 2-[(1*RS*,3*RS*,3a*RS*,6a*SR*)-5-Benzyl-4,6-dioxo-3- phenyloctahydropyrrolo[3,4-*c*]pyrrol-1-yl]acetamide. Yield 22%, colorless crystals, mp 148–149°C. ^1^H NMR (DMSO-d_6_, δ, *J*/Hz): 2.44 (dd, 1H, *J* = 15.0, 8.8); 2.58 (dd, 1H, *J* = 15.0, 3.5); 3.23 (dd, 1H, *J* = 8.8, 7.5); 3.42 (dt, 2H, *J* = 8.1, 3.5); 4.15 (d, 1H, *J* = 7.5); 4.57 (s, 2H); 6.93 (s, 1H); 7.25–7.31(m, 4H); 7.32–7.40 (m, 5H); 7.46 (br.s, 1H); 7.47–7.51 (m, 2H). Found (%): C, 69.34; H, 5.85; N, 11.78. C_21_H_21_N_3_O_3_. Calculated (%): C, 69.41; H, 5.82; N, 11.56. The crystals for X-ray analysis were obtained by slow evaporation of saturated chloroform solution at ambient tempreture.

### Refinement

All hydrogen atoms were located in a difference Fourier map and refined with isotropic thermal parameters.

### Crystal data

**Table:**

C_21_H_21_N_3_O_3_	*Z* = 2
*M**_r_* = 363.41	*F*(000) = 384
Triclinic, *P*1	*D*_x_ = 1.260 Mg m^−^^3^
Hall symbol: -P 1	Mo *K*α radiation, λ = 0.71073 Å
*a* = 9.101 (5) Å	Cell parameters from 25 reflections
*b* = 9.270 (5) Å	θ = 12–13°
*c* = 12.945 (5) Å	µ = 0.09 mm^−^^1^
α = 103.73 (4)°	*T* = 293 K
β = 92.30 (4)°	Block, colourless
γ = 113.94 (4)°	0.50 × 0.40 × 0.30 mm
*V* = 958.1 (8) Å^3^	

### Data collection

**Table:**

Enraf–Nonius CAD-4 diffractometer	*R*_int_ = 0.011
Radiation source: fine-focus sealed tube	θ_max_ = 25.5°, θ_min_ = 2.5°
graphite	*h* = −10→10
ω scans	*k* = −11→11
4399 measured reflections	*l* = −2→15
3561 independent reflections	2 standard reflections every 120 min
2309 reflections with *I* > 2σ(*I*)	intensity decay: none

### Refinement

**Table:**

Refinement on *F*^2^	Secondary atom site location: difference Fourier map
Least-squares matrix: full	Hydrogen site location: difference Fourier map
*R*[*F*^2^ > 2σ(*F*^2^)] = 0.035	All H-atom parameters refined
*wR*(*F*^2^) = 0.108	*w* = 1/[σ^2^(*F*_o_^2^) + (0.0636*P*)^2^ + 0.0755*P*] where *P* = (*F*_o_^2^ + 2*F*_c_^2^)/3
*S* = 1.02	(Δ/σ)_max_ < 0.001
3561 reflections	Δρ_max_ = 0.23 e Å^−^^3^
329 parameters	Δρ_min_ = −0.16 e Å^−^^3^
0 restraints	Extinction correction: *SHELXTL* (Sheldrick, 2008), Fc^*^=kFc[1+0.001xFc^2^λ^3^/sin(2θ)]^-1/4^
Primary atom site location: structure-invariant direct methods	Extinction coefficient: 0.019 (3)

### Special details

**Table:**

Geometry. All e.s.d.'s (except the e.s.d. in the dihedral angle between two l.s. planes) are estimated using the full covariance matrix. The cell e.s.d.'s are taken into account individually in the estimation of e.s.d.'s in distances, angles and torsion angles; correlations between e.s.d.'s in cell parameters are only used when they are defined by crystal symmetry. An approximate (isotropic) treatment of cell e.s.d.'s is used for estimating e.s.d.'s involving l.s. planes.
Refinement. Refinement of *F*^2^ against ALL reflections. The weighted *R*-factor *wR* and goodness of fit *S* are based on *F*^2^, conventional *R*-factors *R* are based on *F*, with *F* set to zero for negative *F*^2^. The threshold expression of *F*^2^ > σ(*F*^2^) is used only for calculating *R*-factors(gt) *etc*. and is not relevant to the choice of reflections for refinement. *R*-factors based on *F*^2^ are statistically about twice as large as those based on *F*, and *R*- factors based on ALL data will be even larger.

### Fractional atomic coordinates and isotropic or equivalent isotropic displacement parameters (Å^2^)

**Table:**

	*x*	*y*	*z*	*U*_iso_*/*U*_eq_	
N1	−0.24934 (15)	0.58343 (15)	0.22990 (10)	0.0479 (3)	
N2	0.22129 (15)	0.92640 (17)	0.28729 (10)	0.0483 (3)	
N3	0.32943 (19)	1.0559 (2)	−0.00733 (12)	0.0609 (4)	
O1	−0.27577 (14)	0.69214 (15)	0.09260 (9)	0.0641 (3)	
O2	−0.16268 (15)	0.53957 (17)	0.38310 (11)	0.0802 (4)	
O3	0.36090 (17)	0.8890 (2)	0.08216 (11)	0.0822 (5)	
C1	0.15214 (17)	0.83233 (18)	0.36351 (11)	0.0422 (3)	
C2	−0.02806 (17)	0.80608 (18)	0.34669 (11)	0.0425 (3)	
C3	−0.15032 (18)	0.6298 (2)	0.32723 (12)	0.0504 (4)	
C4	−0.20472 (17)	0.70517 (18)	0.17794 (11)	0.0441 (3)	
C5	−0.05359 (16)	0.84700 (18)	0.24194 (11)	0.0399 (3)	
C6	0.10000 (17)	0.85783 (19)	0.19148 (11)	0.0419 (3)	
C7	0.13948 (19)	0.9617 (2)	0.11336 (14)	0.0480 (4)	
C8	0.28660 (18)	0.9660 (2)	0.06133 (12)	0.0504 (4)	
C9	−0.3871 (2)	0.4207 (2)	0.18643 (16)	0.0583 (4)	
C10	−0.54238 (19)	0.41544 (18)	0.22548 (13)	0.0510 (4)	
C11	−0.6492 (2)	0.4469 (2)	0.16611 (15)	0.0647 (5)	
C12	−0.7917 (3)	0.4421 (3)	0.2033 (2)	0.0843 (7)	
C13	−0.8263 (3)	0.4063 (3)	0.2991 (2)	0.0888 (7)	
C14	−0.7207 (3)	0.3752 (3)	0.3577 (2)	0.0867 (7)	
C15	−0.5794 (3)	0.3789 (2)	0.32126 (16)	0.0691 (5)	
C16	0.23862 (16)	0.91778 (18)	0.47794 (11)	0.0410 (3)	
C17	0.34576 (19)	1.0818 (2)	0.50956 (13)	0.0510 (4)	
C18	0.4222 (2)	1.1578 (2)	0.61503 (15)	0.0604 (4)	
C19	0.3912 (2)	1.0709 (3)	0.69041 (14)	0.0607 (5)	
C20	0.2857 (2)	0.9079 (3)	0.65957 (14)	0.0651 (5)	
C21	0.2094 (2)	0.8303 (2)	0.55396 (13)	0.0559 (4)	
H1	0.1496 (17)	0.7255 (19)	0.3441 (11)	0.043 (4)*	
H6	0.0843 (17)	0.7458 (19)	0.1533 (11)	0.045 (4)*	
H72	0.045 (2)	0.917 (2)	0.0574 (14)	0.060 (5)*	
H5	−0.0619 (17)	0.9502 (18)	0.2500 (11)	0.041 (4)*	
H22	−0.0542 (19)	0.874 (2)	0.4050 (13)	0.054 (4)*	
H17	0.369 (2)	1.143 (2)	0.4586 (14)	0.062 (5)*	
H71	0.158 (2)	1.071 (2)	0.1478 (14)	0.063 (5)*	
H21	0.135 (2)	0.718 (2)	0.5356 (14)	0.068 (5)*	
H92	−0.356 (2)	0.339 (2)	0.2133 (14)	0.070 (5)*	
H18	0.497 (2)	1.268 (2)	0.6354 (15)	0.075 (6)*	
H91	−0.397 (2)	0.400 (2)	0.1100 (16)	0.072 (6)*	
H2	0.308 (2)	0.920 (2)	0.2746 (13)	0.060 (5)*	
H19	0.450 (2)	1.126 (2)	0.7648 (17)	0.083 (6)*	
H32	0.275 (2)	1.114 (2)	−0.0222 (15)	0.074 (6)*	
H11	−0.622 (2)	0.479 (2)	0.1011 (16)	0.076 (6)*	
H15	−0.502 (3)	0.348 (3)	0.3604 (17)	0.099 (7)*	
H20	0.262 (2)	0.843 (2)	0.7129 (17)	0.085 (6)*	
H31	0.423 (3)	1.069 (2)	−0.0340 (16)	0.082 (6)*	
H14	−0.748 (3)	0.347 (3)	0.4305 (19)	0.111 (8)*	
H12	−0.858 (3)	0.462 (3)	0.1610 (19)	0.103 (8)*	
H13	−0.926 (3)	0.398 (3)	0.323 (2)	0.111 (8)*	

### Atomic displacement parameters (Å^2^)

**Table:**

	*U*^11^	*U*^22^	*U*^33^	*U*^12^	*U*^13^	*U*^23^
N1	0.0463 (7)	0.0459 (7)	0.0468 (7)	0.0128 (6)	0.0111 (6)	0.0166 (6)
N2	0.0359 (7)	0.0718 (9)	0.0450 (7)	0.0238 (6)	0.0155 (5)	0.0272 (6)
N3	0.0601 (9)	0.0864 (11)	0.0618 (9)	0.0404 (8)	0.0319 (7)	0.0461 (8)
O1	0.0597 (7)	0.0739 (8)	0.0508 (7)	0.0174 (6)	0.0008 (5)	0.0249 (6)
O2	0.0680 (8)	0.0850 (9)	0.0834 (9)	0.0099 (7)	0.0072 (7)	0.0579 (8)
O3	0.0839 (9)	0.1354 (12)	0.0919 (10)	0.0783 (9)	0.0596 (8)	0.0819 (9)
C1	0.0432 (8)	0.0473 (9)	0.0417 (8)	0.0220 (7)	0.0146 (6)	0.0164 (7)
C2	0.0403 (8)	0.0511 (8)	0.0374 (8)	0.0194 (7)	0.0138 (6)	0.0138 (7)
C3	0.0455 (8)	0.0585 (9)	0.0492 (9)	0.0169 (7)	0.0150 (7)	0.0268 (8)
C4	0.0425 (8)	0.0514 (8)	0.0413 (8)	0.0204 (7)	0.0123 (6)	0.0166 (7)
C5	0.0391 (7)	0.0436 (8)	0.0435 (8)	0.0205 (6)	0.0150 (6)	0.0172 (6)
C6	0.0415 (8)	0.0505 (9)	0.0424 (8)	0.0240 (7)	0.0171 (6)	0.0191 (7)
C7	0.0434 (9)	0.0611 (10)	0.0488 (9)	0.0246 (8)	0.0156 (7)	0.0261 (8)
C8	0.0497 (9)	0.0702 (10)	0.0435 (8)	0.0296 (8)	0.0173 (7)	0.0288 (8)
C9	0.0572 (10)	0.0448 (9)	0.0604 (11)	0.0109 (8)	0.0126 (8)	0.0115 (8)
C10	0.0520 (9)	0.0370 (8)	0.0525 (9)	0.0090 (7)	0.0098 (7)	0.0101 (7)
C11	0.0676 (11)	0.0618 (11)	0.0554 (10)	0.0225 (9)	0.0022 (9)	0.0105 (9)
C12	0.0716 (14)	0.0691 (13)	0.1083 (18)	0.0337 (11)	−0.0010 (13)	0.0137 (12)
C13	0.0777 (15)	0.0622 (12)	0.130 (2)	0.0300 (11)	0.0492 (15)	0.0267 (13)
C14	0.0970 (17)	0.0768 (14)	0.0990 (17)	0.0351 (13)	0.0525 (14)	0.0445 (13)
C15	0.0751 (12)	0.0611 (11)	0.0751 (13)	0.0222 (9)	0.0264 (10)	0.0363 (10)
C16	0.0377 (7)	0.0511 (8)	0.0428 (8)	0.0245 (6)	0.0125 (6)	0.0170 (6)
C17	0.0521 (9)	0.0527 (9)	0.0531 (9)	0.0243 (8)	0.0122 (7)	0.0192 (8)
C18	0.0535 (10)	0.0590 (11)	0.0627 (11)	0.0251 (9)	0.0055 (8)	0.0048 (9)
C19	0.0530 (10)	0.0871 (14)	0.0468 (9)	0.0407 (10)	0.0049 (8)	0.0080 (9)
C20	0.0699 (12)	0.0902 (14)	0.0494 (10)	0.0406 (11)	0.0139 (9)	0.0321 (10)
C21	0.0582 (10)	0.0600 (11)	0.0508 (9)	0.0211 (9)	0.0101 (8)	0.0250 (8)

### Geometric parameters (Å, °)

**Table:**

N1—C4	1.379 (2)	C9—C10	1.506 (2)
N1—C3	1.386 (2)	C9—H92	1.030 (18)
N1—C9	1.473 (2)	C9—H91	0.955 (19)
N2—C6	1.452 (2)	C10—C11	1.377 (3)
N2—C1	1.4639 (19)	C10—C15	1.381 (2)
N2—H2	0.832 (18)	C11—C12	1.389 (3)
N3—C8	1.324 (2)	C11—H11	0.97 (2)
N3—H32	0.91 (2)	C12—C13	1.374 (4)
N3—H31	0.90 (2)	C12—H12	0.90 (2)
O1—C4	1.2120 (18)	C13—C14	1.361 (4)
O2—C3	1.2053 (19)	C13—H13	0.95 (3)
O3—C8	1.2281 (19)	C14—C15	1.378 (3)
C1—C16	1.511 (2)	C14—H14	1.05 (2)
C1—C2	1.555 (2)	C15—H15	1.01 (2)
C1—H1	0.953 (15)	C16—C17	1.378 (2)
C2—C3	1.509 (2)	C16—C21	1.386 (2)
C2—C5	1.525 (2)	C17—C18	1.384 (3)
C2—H22	0.966 (16)	C17—H17	0.943 (18)
C4—C5	1.494 (2)	C18—C19	1.375 (3)
C5—C6	1.543 (2)	C18—H18	0.93 (2)
C5—H5	0.972 (14)	C19—C20	1.368 (3)
C6—C7	1.513 (2)	C19—H19	0.99 (2)
C6—H6	0.987 (15)	C20—C21	1.386 (3)
C7—C8	1.513 (2)	C20—H20	1.00 (2)
C7—H72	0.978 (18)	C21—H21	0.944 (19)
C7—H71	0.944 (19)		
			
C4—N1—C3	112.94 (13)	O3—C8—N3	122.40 (15)
C4—N1—C9	123.65 (14)	O3—C8—C7	121.10 (13)
C3—N1—C9	123.41 (14)	N3—C8—C7	116.50 (14)
C6—N2—C1	107.05 (12)	N1—C9—C10	112.20 (14)
C6—N2—H2	112.4 (12)	N1—C9—H92	106.3 (10)
C1—N2—H2	109.2 (12)	C10—C9—H92	109.0 (10)
C8—N3—H32	122.6 (12)	N1—C9—H91	105.6 (11)
C8—N3—H31	118.2 (12)	C10—C9—H91	111.2 (11)
H32—N3—H31	118.8 (17)	H92—C9—H91	112.5 (15)
N2—C1—C16	113.54 (13)	C11—C10—C15	119.19 (18)
N2—C1—C2	101.61 (11)	C11—C10—C9	120.46 (16)
C16—C1—C2	114.38 (12)	C15—C10—C9	120.35 (17)
N2—C1—H1	112.9 (9)	C10—C11—C12	119.8 (2)
C16—C1—H1	108.1 (8)	C10—C11—H11	120.2 (12)
C2—C1—H1	106.1 (9)	C12—C11—H11	119.9 (12)
C3—C2—C5	104.30 (13)	C13—C12—C11	120.3 (2)
C3—C2—C1	114.00 (13)	C13—C12—H12	123.5 (16)
C5—C2—C1	106.24 (11)	C11—C12—H12	116.2 (16)
C3—C2—H22	108.6 (9)	C14—C13—C12	119.9 (2)
C5—C2—H22	110.1 (9)	C14—C13—H13	120.0 (15)
C1—C2—H22	113.2 (9)	C12—C13—H13	120.1 (15)
O2—C3—N1	123.89 (15)	C13—C14—C15	120.3 (2)
O2—C3—C2	127.97 (15)	C13—C14—H14	119.8 (13)
N1—C3—C2	108.13 (13)	C15—C14—H14	119.9 (13)
O1—C4—N1	124.11 (15)	C14—C15—C10	120.5 (2)
O1—C4—C5	127.43 (14)	C14—C15—H15	122.1 (12)
N1—C4—C5	108.40 (13)	C10—C15—H15	117.3 (12)
C4—C5—C2	105.30 (12)	C17—C16—C21	118.60 (15)
C4—C5—C6	112.59 (12)	C17—C16—C1	121.77 (14)
C2—C5—C6	103.62 (11)	C21—C16—C1	119.62 (14)
C4—C5—H5	111.1 (8)	C16—C17—C18	120.74 (17)
C2—C5—H5	115.4 (8)	C16—C17—H17	119.8 (11)
C6—C5—H5	108.6 (8)	C18—C17—H17	119.4 (11)
N2—C6—C7	113.43 (13)	C19—C18—C17	120.39 (18)
N2—C6—C5	100.79 (11)	C19—C18—H18	119.3 (12)
C7—C6—C5	113.69 (12)	C17—C18—H18	120.3 (12)
N2—C6—H6	111.2 (8)	C20—C19—C18	119.24 (17)
C7—C6—H6	108.8 (8)	C20—C19—H19	121.2 (12)
C5—C6—H6	108.7 (8)	C18—C19—H19	119.5 (12)
C6—C7—C8	113.10 (13)	C19—C20—C21	120.81 (18)
C6—C7—H72	108.4 (9)	C19—C20—H20	120.4 (12)
C8—C7—H72	109.2 (10)	C21—C20—H20	118.8 (12)
C6—C7—H71	111.0 (10)	C20—C21—C16	120.22 (18)
C8—C7—H71	108.4 (11)	C20—C21—H21	118.8 (11)
H72—C7—H71	106.6 (14)	C16—C21—H21	121.0 (11)
			
C6—N2—C1—C16	−162.41 (12)	C4—C5—C6—C7	90.44 (16)
C6—N2—C1—C2	−39.10 (14)	C2—C5—C6—C7	−156.31 (13)
N2—C1—C2—C3	129.33 (13)	N2—C6—C7—C8	67.80 (18)
C16—C1—C2—C3	−107.94 (15)	C5—C6—C7—C8	−177.81 (13)
N2—C1—C2—C5	15.04 (14)	C6—C7—C8—O3	0.9 (2)
C16—C1—C2—C5	137.77 (13)	C6—C7—C8—N3	−179.38 (15)
C4—N1—C3—O2	−176.19 (15)	C4—N1—C9—C10	−92.44 (19)
C9—N1—C3—O2	3.4 (2)	C3—N1—C9—C10	87.97 (19)
C4—N1—C3—C2	2.69 (17)	N1—C9—C10—C11	92.8 (2)
C9—N1—C3—C2	−177.68 (13)	N1—C9—C10—C15	−87.3 (2)
C5—C2—C3—O2	171.11 (16)	C15—C10—C11—C12	0.2 (3)
C1—C2—C3—O2	55.7 (2)	C9—C10—C11—C12	−179.83 (16)
C5—C2—C3—N1	−7.71 (15)	C10—C11—C12—C13	0.1 (3)
C1—C2—C3—N1	−123.14 (13)	C11—C12—C13—C14	−0.1 (3)
C3—N1—C4—O1	−178.84 (14)	C12—C13—C14—C15	−0.2 (4)
C9—N1—C4—O1	1.5 (2)	C13—C14—C15—C10	0.5 (3)
C3—N1—C4—C5	3.74 (16)	C11—C10—C15—C14	−0.5 (3)
C9—N1—C4—C5	−175.89 (13)	C9—C10—C15—C14	179.51 (17)
O1—C4—C5—C2	174.29 (15)	N2—C1—C16—C17	13.95 (19)
N1—C4—C5—C2	−8.41 (15)	C2—C1—C16—C17	−102.06 (16)
O1—C4—C5—C6	−73.49 (19)	N2—C1—C16—C21	−166.52 (13)
N1—C4—C5—C6	103.82 (14)	C2—C1—C16—C21	77.47 (17)
C3—C2—C5—C4	9.55 (14)	C21—C16—C17—C18	−0.2 (2)
C1—C2—C5—C4	130.31 (12)	C1—C16—C17—C18	179.33 (13)
C3—C2—C5—C6	−108.87 (13)	C16—C17—C18—C19	−0.6 (2)
C1—C2—C5—C6	11.89 (14)	C17—C18—C19—C20	0.9 (3)
C1—N2—C6—C7	168.92 (12)	C18—C19—C20—C21	−0.4 (3)
C1—N2—C6—C5	47.03 (14)	C19—C20—C21—C16	−0.4 (3)
C4—C5—C6—N2	−147.85 (12)	C17—C16—C21—C20	0.7 (2)
C2—C5—C6—N2	−34.60 (14)	C1—C16—C21—C20	−178.86 (14)

### Hydrogen-bond geometry (Å, °)

**Table:**

Cg1 is the centroid of the C10–C15 phenyl ring.

**Table:**

*D*—H···*A*	*D*—H	H···*A*	*D*···*A*	*D*—H···*A*
N3—H32···O1^i^	0.91 (2)	2.194 (19)	3.003 (2)	147.3 (16)
N3—H31···O3^ii^	0.90 (2)	2.00 (2)	2.903 (2)	175.7 (18)
C18—H18···Cg1^iii^	0.93 (2)	2.71	3.627	166.5

Symmetry codes: (i) −*x*, −*y*+2, −*z*; (ii) −*x*+1, −*y*+2, −*z*; (iii) −*x*, −*y*+2, −*z*+1.
